# Anterior Lateral Thigh Perforator (ALTP) Flaps: Literature Review and Technical Experience to Pushing the Limits Toward Aesthetical Reconstruction

**DOI:** 10.3390/medicina61122154

**Published:** 2025-12-03

**Authors:** Ziyad Alharbi, Sarah Qari, Tala Zafar, Faris Almarzouqi, Benedikt Schaefer, Johannes Hertelendy, Savas Tsolakidis, Anas Fathuldeen, Hans-Oliver Rennekampff

**Affiliations:** 1 Plastic Surgery and Burn Unit, Dr. Soliman Fakeeh Hospital, Jeddah 23323, Saudi Arabia; zialharbi@fakeeh.care (Z.A.); sarahyqari@gmail.com (S.Q.); 2 Clinical Sciences Department, Fakeeh College for Medical Sciences, Jeddah 23323, Saudi Arabia; 3 College of Medicine, King Abdulaziz University (KAU), Jeddah 21589, Saudi Arabia; 4 Department of Plastic Surgery, Dr. Sulaiman Al-Habib Hospital, Jeddah 22245, Saudi Arabia; faris.almarzouqi@gmail.com; 5 Department of Plastic Surgery and Burn Unit, RWTH Aachen University, 52062 Aachen, Germany; bschaefer@ukaachen.de; 6 Department of Plastic Surgery, Kaiserswerther Diakonie Florence-Nightingale-Krankenhaus, 40489 Düsseldorf, Germany; johannes.hertelendy@t-online.de; 7 Austrian Cluster of Tissue Regeneration and Ludwig Boltzmann Institute for Experimental and Clinical Traumatology, Research Centre for Traumatology of the Austrian Workers’ Compensation Board (AUVA), Donaueschingenstraße 13, 1200 Vienna, Austria; s.tsolakidis@me.com; 8 College of Medicine, Hail University, Hail 55476, Saudi Arabia; afathuldeen@hotmail.com; 9 Department of Plastic and Aesthetic Surgery, Burn Surgery, Rhein-Maas Klinikum, 52146 Würselen, Germany; hans-oliver.rennekampff@rheinmaasklinikum.de

**Keywords:** ankle, anterior lateral thigh perforator flaps, extremity, fasciocutaneous flaps, foot, free flaps, reconstruction

## Abstract

*Background and Objectives*: Free tissue transfer for coverage of large defects is a common technique in plastic surgery. The kind of free tissue transfer depends on various factors such as the size of the defect, type and content of missing tissue, the location, and the weight-bearing demands of the area. Instead of performing bulky muscle free flaps, another alternative has bas been considered. *Materials and Methods*: This is a technical experience report for ALTP Flap Surgery conducted from 2013 to 2017 which included 15 surgery reviews out of 50 based on the inclusion criteria to identify the result of aesthetical reconstruction and to include such results with the current literature. In addition to that, a narrative review of the literature has been performed using PubMed and Google Scholar by using the ALT or ALTP Flap terms to determine the current practice and to compare with our results. *Results*: Single-stage debridement of all defects was followed by reconstruction in all surgeries. A total of 14 Flaps succeeded (93.3%) without any complications and only one of the flaps was complicated (6.7%) with vein thrombosis which resulted in the loss of that flap, leading to another revision being performed for that particular patient. No hematoma or infection has been noted. *Conclusions*: Using an extended-size ALTP flap can be a reliable option for the reconstruction of the weight-bearing area of the foot and offers a good postoperative function and esthetic result without the need of further subsequent debulking procedures.

## 1. Introduction

Free tissue transfer for coverage of large defects is a common technique in plastic surgery. The kind of free tissue transfer depends on various factors such as the size of the defect, type and content of missing tissue, the location, and the weight-bearing demands of the area. Instead of performing bulky muscle free flaps, another alternative has been considered.

Soft tissue defects in the extremities present a significant challenge in reconstructive surgery for orthopedic and plastic surgeons, often requiring innovative approaches to achieve maximal functional and esthetic outcomes [1,2,3,4]. For such deformities, free perforator skin flaps are the ideal choice. The literature lists a variety of free flap options, including the medial sural artery perforator (MSAP) flap known for its thin, pliable skin, low donor site morbidity, and is preferred when smaller flaps are needed [5]. Additionally, the deep inferior epigastric perforator (DIEP) flap provides abundant soft tissue taken from the lower abdomen sparing the rectus muscle, unlike the transverse rectus abdominis myocutaneous flap (TRAM flaps) where both flaps are mainly used in breast reconstruction [6]. The anterolateral thigh perforator (ALTP) flap has transformed reconstructive surgery by providing a versatile, dependable, and customizable solution for treating complex abnormalities in the head and neck, axilla, and extremities. Its broad skin paddle, long vascular pedicle, and minimal impact on function and appearance at the donor site make it ideal for lower limb restoration, including the foot and ankle [7].

The approach to harvesting soft tissue free flaps has evolved considerably over the past several decades. Musculocutaneous free flap was the first type to be developed which included both the skin and the underlying muscle; however, it has presented with higher rates of donor site morbidity and debulking. In 1981, Pontén introduced the fasciocutaneous flap which included the cutaneous, subcutaneous tissue and the deep muscle fascia while preserving the underlying muscle in a subfascial plane. The is known to be the traditional approach, yet still resulted in thick flaps, potential contour deformities, and increased donor site morbidity, sometimes necessitating secondary debulking procedures [8]. The suprafascial harvesting technique for the anterolateral thigh (ALT) flap was introduced to address the limitations of the traditional subfascial approach. This includes the skin and subcutaneous tissue while preserving the fascia and muscle, providing a thinner flap, therefore preserving donor site structure, reducing contour irregularities, and permitting primary closure in all cases, leading to higher patient satisfaction with better esthetic contour and less need for secondary debulking [9].

This retrospective study aims to evaluate the benefits of using ATLP flaps in the reconstruction of soft tissue defects in the extremities, including outcomes related to flap survival, functional and esthetic restoration, as well as patient satisfaction. By analyzing a series of cases treated with ATLP flaps, this study seeks to provide valuable insights into the efficacy and advantages of this surgical technique in improving patients’ outcomes.

## 2. Materials and Methods

This is a technical experience and clinical presentation report for ALTP Flap Surgery conducted from 2013 to 2017 which included 15 surgery reviews out of 50 based on the inclusion criteria to identify the result of esthetical reconstruction and to include such results with the current literature. We identified these 15 flap surgeries based on the location, foot and ankle, and excluded the legs, knee and thigh region as well as the upper limb area. We also excluded any comorbidities that might affect the course of the flap procedure including pediatric group. The retrospective review focused on describing our surgical technique, flap survival, complications, and esthetic outcomes, providing illustrative examples of our operative approach. In addition, a narrative review of the literature has been performed through PubMed and Google Scholar using the terms “ALT” or “ALTP Flap”. Relevant studies were identified, allowing us to summarize current clinical practices and outcomes of ALTP flaps which were then compared with our results. This review highlighted the surgical practices with a comparison to the current literature review (Table 1).

**Figure 1:**
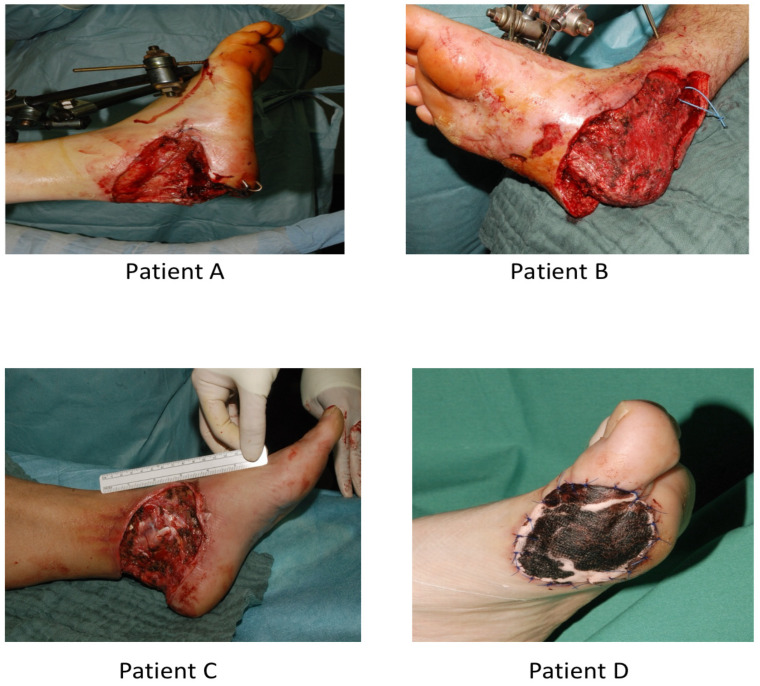
Initial debridement of 4 different patients prior to ALTP flap coverage in the same operative session.

With every patient, initial debridement was immediately followed by ALTP flap coverage in a single operative session, as shown in Figure 1; the plastic surgery team was to evaluate each case for defect reconstruction during the hospital stay of the patient.

The skin and subcutaneous tissue along with the medial border of the flap is incised down to the level of the facia lata, revealing the rectus femoris muscle underneath. In most cases, a myocutaneous perforator will be found traversing the medial edge of the vastus lateralis muscle. However, in some, a septocutaneous perforator is found in the intermuscular septum between the rectus femoris and vastus lateralis muscle, and rarely both perforators are present.

Once the flap’s perforators are identified and its course to the descending branch of the lateral circumflex femoral artery is confirmed, the flap’s lateral border can be incised, and the fascia can be transected in the proximal and distal borders while maintaining a safe distance of 2–3 cm from the perforators.

The vastus lateralis muscle is dissected to release the myocutaneous perforator, leaving a small muscle cuff to identify the direction of the vessel and prevent twisting and damage. In the case of a septocutaneous perforator, the pedicle is easily twisted since the skin vessel lacks the muscle to maintain appropriate orientation. To avoid pedicle twisting, a fascia cuff is preserved around the pedicle. Special attention is given to preserving the motor branch of the femoral nerve to the vastus lateralis muscle.

The flap will then be raised, and the proximal end of the pedicle will be divided distal to the branch of the rectus femoral muscle to preserve its blood supply. Hemostasis was meticulously performed using a bipolar cautery.

When dissecting the flap, the choice between a suprafascial or subfascial dissection is crucial and depends on the recipient site needs and the acceptable level of donor site morbidity for the individual patient. Suprafascial dissection is preferred for thin flaps where donor site morbidity reduction and sensory innervation preservation are required [10]. The subfascial approach facilitates the identification of skin vessels and provides better exposure of the intermuscular septum and descending branch of the LCFA, giving the surgeon a clear vascular anatomy map before proceeding with dissection of the pedicle. We used suprafascial dissection to preserve the deep fascia at the donor site, thereby maintaining structural integrity, minimizing contour deformities, and allowing for primary closure in all cases, which contributed to higher patient satisfaction. This technique also provides thinner and more versatile flaps with improved esthetic contouring, minimizing the need for secondary debulking procedures.

The type used was an adipocutaneous flap with suprafascial dissection in order to provide more support for the donor site area, of which all donor sites have been closed with primary closure techniques which resulted in more patient satisfaction.

A portable Doppler probe was utilized to map out the perforators and intraoperative testing of the individual perforators by pinching with bulldog clamps showed good perfusion for the flap. With the patient in a supine neutral position, a line is first drawn from the anterior superior iliac spine to the superior lateral border of the patella. The midpoint of the line is then marked, and a circle with a 3 cm radius is drawn. The majority of perforators are in the circle’s inferior lateral quadrant. The perforator does not need to be in the center of the flap. The flap can be built with the perforator off-center if a longer pedicle is needed.

**Figure 2:**
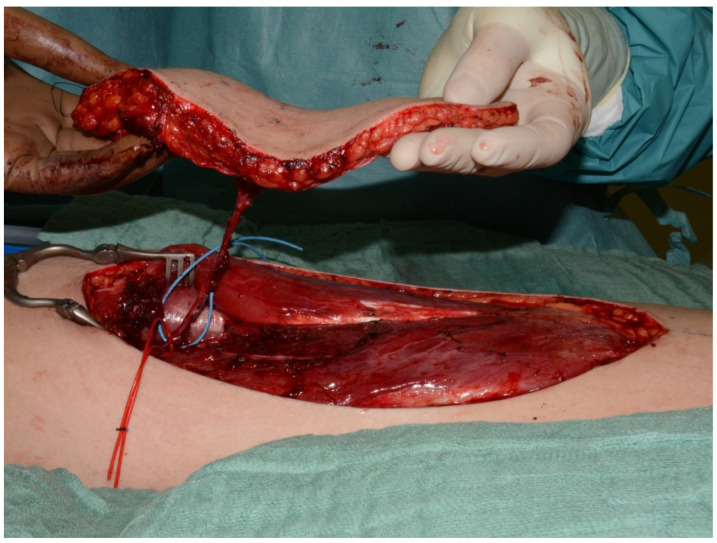
Preparation of recipient vessels and end-to-side anastomosis technique.

ALTP flaps were raised in different sizes ranging from 12 × 7 cm up to 31 × 13 cm depending on the defect size of each patient. Each flap included either 2 or 3 perforators as branches of the descending branch of the lateral circumflex femoral artery (Figure 2).

**Figure 3:**
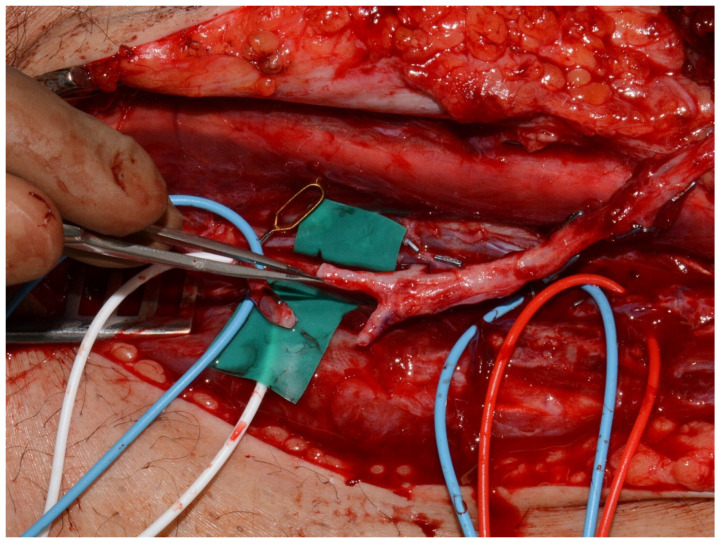
The ALTP-Flap and the vessels after complete dissection.

We planned a closure of the defects through ALTP Flaps for both upper and lower limbs using different recipient vessels: radial or ulnar in upper limbs or posterior or anterior tibial through end-to-side anastomosis technique in lower limbs as shown in Figure 3.

## 3. Results

Single-stage debridement of all defects was followed by reconstruction in all surgeries. A total of 14 Flaps succeeded (93.3%) without any complications and only one out of the 15 flaps were complicated with vein thrombosis (6.7%) which resulted in loss of the flap, leading to another revision being performed for that particular flap. No hematoma or infection has been noted.

**Figure 4:**
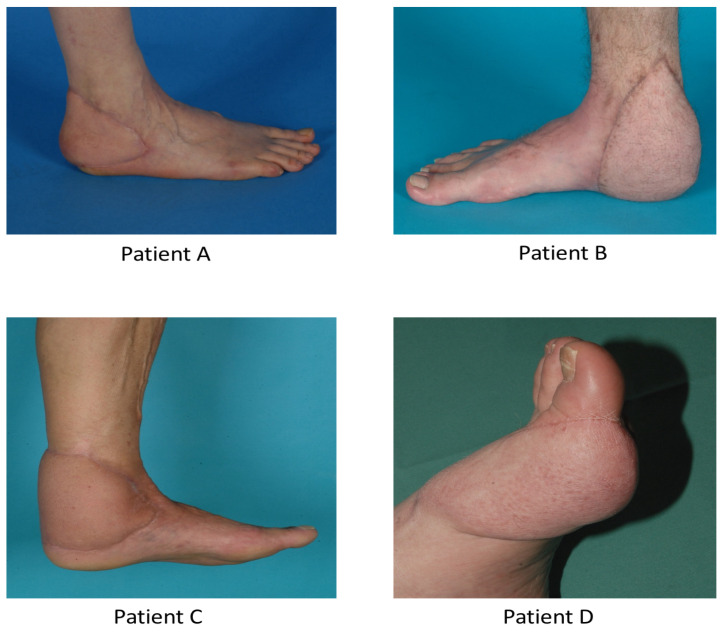
Complete flap coverage of the same 4 patients 18 months after surgery showing optimal reconstructive and esthetic result.

The postoperative status was stable. The patency of the anastomosis was judged clinically as well as by Doppler device. The flaps were completely perfused at each point in time. In particular, there was no necrosis of the end of the flap. A complete take rate was noticeable for all flaps in the upper limb area as shown in Figure 4. The flaps were also completely perfused at each point in time for the lower limb area.

## 4. Discussion

When choosing the flap type, it is important to analyze the recipient site precisely for a successful tissue transfer [11]. Reconstruction by free tissue transfer is technically challenging and demands a high expertise of the operating staff. In these cases, the whole foot, in particular the weight-bearing area of the foot sole, has to be covered. The preservation of the foot and the rehabilitation of the foot’s function were the main goals of treatment. We decided to use ALTP flaps for reconstruction of the weight-bearing area [12]. Numerous papers discuss the use of fasciocutaneous, musculocutaneous, and split skin transplanted muscle flaps for foot sole restoration.

The findings of this retrospective study highlight the significant benefits of utilizing anterolateral thigh perforator (ATLP) flaps for repairing soft tissue abnormalities in the extremities. Our results demonstrate favorable outcomes in terms of flap survival rates, functional restoration, and patient satisfaction, reaffirming the efficacy and versatility of ATLP flaps in addressing complex defects in this anatomical region. The ability of ATLP flaps to provide reliable vascularized tissue coverage, along with their versatility in accommodating various defect sizes and shapes, makes them a valuable tool for reconstruction surgeons.

The two main free flaps used in soft tissue reconstruction are the ALTP and the medial sural artery perforator (MSAP) flaps. When comparing the two flaps, the ALTP flap has a long pedicle with adequate vessel diameter, consistent anatomy, and the ability to harvest a large paddle of skin while the MSAP flap is more thin, pliable, and requires less harvest time [13,14]. While both offer minimal sensory and functional deficit, a study conducted by Chakraborty et al. comparing both flaps in head, neck, and extremity reconstruction found them to be similar in esthetic outcome but the ALTP flap was found to be superior to the MSAP flap in terms of pedicle length, vessel diameter, and donor site morbidity, while the latter takes less time for harvest [15,16].

In our practice, ALTP flaps remain the dominant choice as a skin flap option due to the ease of elevation process, anatomical location, and vessel diameter. However, the MSAP flap would be the option if the latter could not be harvested for a particular reason or if the patient preferred a thinner flap [13,14,15,16,17,18,19,20,21,22]. For instance, ALT flaps offer the potential for a two-team strategy to save operating time. The flap donor site can be closed directly if the width is not excessive (our limit has been 9 cm in width for direct closure). It was observed that arteries generally have an appropriate diameter, and that the pedicle is extended enough. Large vessels were preserved during this procedure. Overall, this study’s findings are consistent with ATLP flaps’ continued usage as a reliable and effective option for reconstructing soft tissue defects in the extremities, while highlighting the importance of tailoring flap selection to fit the demands and specific characteristics of each patient. This study is limited by its retrospective design, which restricted the availability of postoperative images for all the cases. Further research and comparative studies may provide additional insights into the optimal use of different flap types in extremity reconstruction, ultimately enhancing patient outcomes and quality of care.

## 5. Conclusions

In cases where a thin and esthetically pleasing soft tissue envelope is needed for the restoration of the foot, ankle, sole, and toes as well as lower limb deformities, a free ALTP flap is a suitable choice. This flap has a better reconstructive and cosmetic outcome, decreased donor site morbidity, and an easier elevation procedure.

## Figures and Tables

**Table 1:** Summary of flap review.

Patient Number	Flap Location	Anastomosis Type	Donor Site Closure	Complications
1	Foot	End to Side	Direct Closure	
2	Foot	End to Side	Direct Closure	-
3	Foot	End to Side	Direct Closure	-
4	Foot	End to Side	Direct Closure	Vein thrombosis which has been revised
5	Foot	End to Side	Direct Closure	-
6	Foot	End to Side	Direct Closure	-
7	Foot	End to Side	Direct Closure	-
8	Foot	End to Side	Direct Closure	-
9	Foot	End to Side	Direct Closure	-
10	Foot	End to Side	Direct Closure	-
11	Foot	End to Side	Direct Closure	-
12	Foot	End to Side	Direct Closure	-
13	Foot	End to Side	Direct Closure	-
14	Foot	End to Side	Direct Closure	-
15	Foot	End to Side	Direct Closure	-

## Data Availability

All data generated or analyzed during this study are included in this published article.

## Abbreviations

The following abbreviations are used in this manuscript:

ALTP	Anterior Lateral Thigh Perforator
LCFA	Lateral Circumflex Femoral Artery
MSAP	Medial Sural Artery Perforator
DIEP	Deep Inferior Epigastric Perforator
TRAM	Transverse Rectus Abdominis Myocutaneous
